# TRPA1 promotes cisplatin-induced acute kidney injury via regulating the endoplasmic reticulum stress-mitochondrial damage

**DOI:** 10.1186/s12967-023-04351-9

**Published:** 2023-10-05

**Authors:** Fei Deng, Heping Zhang, Wei Zhou, Shijie Ma, Yuwei Kang, Wei Yang, Liangbin Zhao, Wei Qin

**Affiliations:** 1 Department of Nephrology, West China Hospital, Sichuan University, 37 Guoxue Alley, Wuhou District, Chengdu, 610044 China; 2 Department of Nephrology, Sichuan Provincial People’s Hospital Jinniu Hospital, Chengdu Jinniu District People’s Hospital, Chengdu, China; 3 Department of Nephrology, Sichuan Provincial People’s Hospital, University of Electronic Science and Technology of China, Chengdu, China; 4 Department of Nephrology, Affiliated Hospital of North Sichuan Medical College, Nanchong, China; 5 Department of Nephrology, Affiliated Hospital of Southwest Medical University, Luzhou, China; 6 Department of Nephrology, Hospital of Chengdu University of Traditional Chinese Medicine, 39 Shierqiao Road, Jinniu District, Chengdu, 610072 China

**Keywords:** Cisplatin, Acute kidney injury, TRPA1, Endoplasmic reticulum stress, Mitochondrial impairment

## Abstract

**Background:**

Cisplatin is a widely used and effective chemotherapeutic agent against cancer. However, nephrotoxicity is one of the most common side effects of cisplatin, and it can proceed to acute kidney injury (AKI). Studies have reported that activation of transient receptor potential ankyrin-1 (TRPA1) mediates cisplatin-induced renal tubular cytotoxic injury. The aim of this study was to investigate the mechanism of TRPA1 in promoting cisplatin-induced AKI through modulation of the endoplasmic reticulum stress (ERS)-mitochondrial damage.

**Methods:**

A cisplatin-induced HK-2 cell model in vitro and mouse model in vivo were established. The mechanism of TRPA1 promotes AKI was elucidated by H&E staining, TUNEL staining, transmission electron microscope (TEM), immunofluorescence, CCK-8 viability assays, flow cytometry, Western blotting, JC-1 assay, and enzyme linked immunosorbent assay (ELISA).

**Result:**

In vivo and in vitro, HC-030031 reduced cisplatin-induced Scr and BUN level elevations; improved cisplatin-induced renal tissue injury, apoptosis, and mitochondrial dysfunction; elevated the reduced ERS-associated proteins glucose-regulated protein 78 (GRP78), glucose-regulated protein 75 (GRP75), and C/EBP homologous protein (CHOP) levels induced by cisplatin; reduced the elevated optic atrophy 1 (OPA1), mito-fusion 1 (MFN1), and mito-fusion 2 (MFN2) protein levels, and elevated phospho-dynamin-related protein 1 (p-DRP1) and mitochondrial fission factor (MFF) protein levels. HC-030031 also reduced the mitochondria-associated endoplasmic reticulum membrane (MAM) structure. In addition, TRPA1 agonists also decreased cell proliferation, increased apoptosis, and triggered mitochondrial dysfunction and calcium overload in HK-2 cells via modulation of MAM. ERS inhibitors and GRP75 inhibitors reversed these changes caused by TRPA1 agonists.

**Conclusion:**

Our findings suggest that TRPA1 enhances cisplatin-induced AKI via modulation of ERS and mitochondrial damage.

## Introduction

**Fig. 1:**
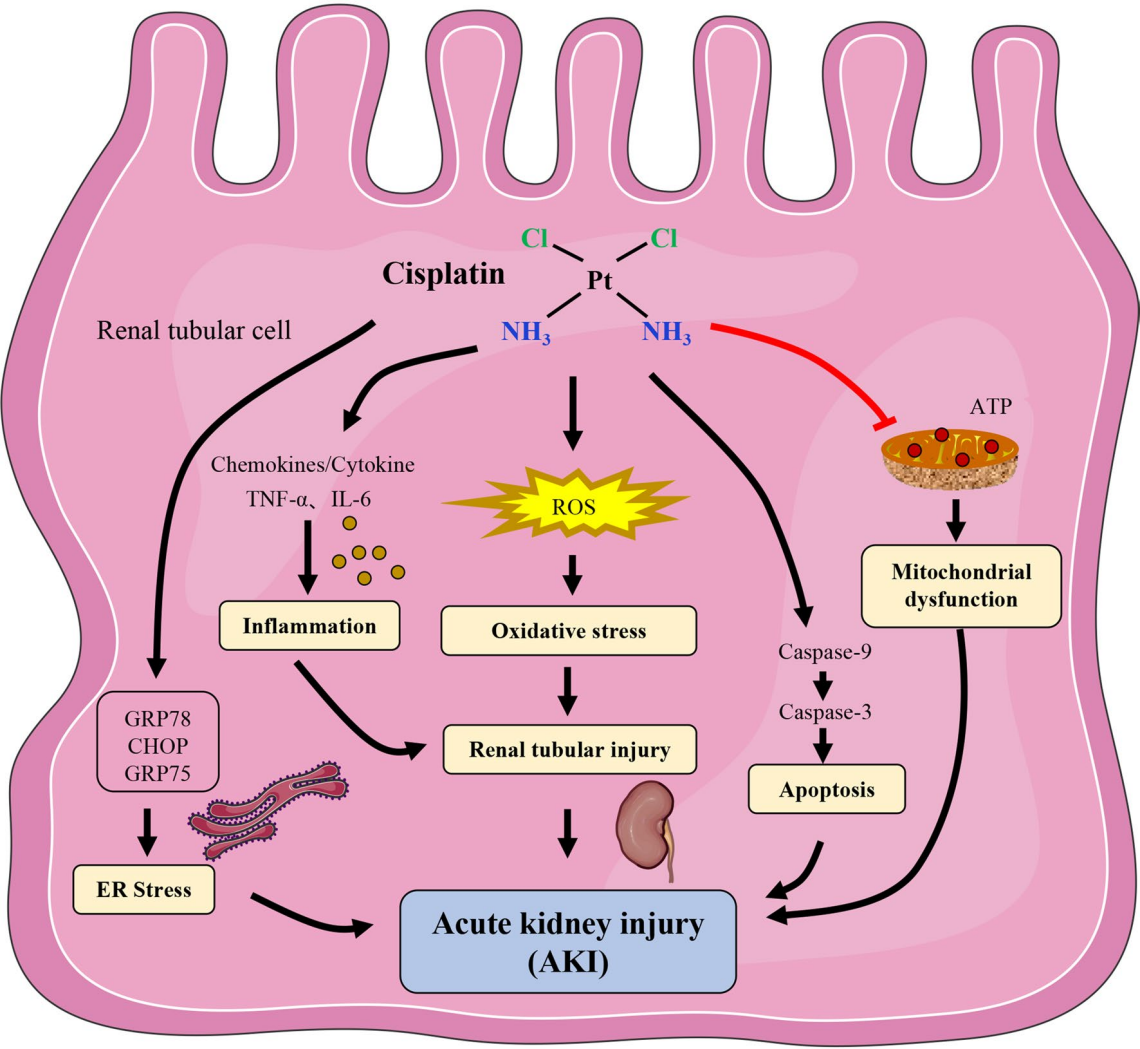
The mechanism of cisplatin-induced acute kidney injury. *ER* endoplasmic reticulum, *ROS* reactive oxygen species, *ATP* adenosine triphosphate

Cisplatin (DDP) is one of the most commonly used and efficient chemotherapy drugs for various types of human cancers, such as testicular, ovarian, bladder, small and non-small cell lung cancer, cervical, sarcoma and lymphoma [1]. Unfortunately, nephrotoxicity, one of the most prominent adverse effects of cisplatin, greatly restricts the therapeutic use of the drug, with 20–30% of cisplatin patients experiencing acute kidney injury (AKI) [2, 3]. AKI is characterized by high morbidity and mortality. However, due to its complex etiology, the pathogenesis of AKI remains unclear (Fig. 1). Therefore, a thorough understanding the molecular pathways and related mechanisms of cisplatin nephrotoxicity is essential for finding or developing drugs suitable for cisplatin combination.

Transient receptor potential ankyrin-1 (TRPA1), a member of the transient receptor potential (TRP) channel family, is a non-selective cation channel with the ability to sense environmental stimuli [4, 5]. It can be activated by a variety of noxious stimuli that increase intracellular Ca^2+^ concentration and Ca^2+^ inward flow, which in turn regulates the occurrence of the corresponding physiological activities or pathogenic processes [6]. Previous studies have demonstrated that TRPA1 channels can be activated by endogenous inflammatory mediators and exogenous noxious stimuli, mediating processes such as inflammation and painful stimulation, suggesting that these channels may be crucial targets for analgesic and anti-inflammatory drugs [7, 8]. Recent studies have pointed out that TRPA1 is engaged in the regulation of kidney cell function [9, 10]. TRPA1 is highly expressed in the renal tubules in AKI patients, which slows the recovery of renal function [11]. Our previous study revealed that cisplatin can promote TRPA1 expression and activation in renal tubular cells and induce cellular Ca^2+^ inward flow leading to cellular Ca^2+^ overload; TRPA1 inhibitor HC-030031 can attenuate cisplatin-induced inflammatory response in renal tubular cells by inhibiting MAPK/NF-κB signaling pathway [12], and also alleviate cisplatin-induced renal tubular cell apoptosis by inhibiting Ca^2+^-dependent signaling pathway [13]. These studies provided evidence that TRPA1 activation facilitates cisplatin-induced AKI and mediates the cytotoxic effects of the drug. However, the molecular mechanism of TRPA1 regulating apoptosis and injury has not been clarified.

Endoplasmic reticulum stress (ERS) and mitochondrial dysfunctional impairment have been extensively highlighted in recent years as contributing factors to cisplatin nephrotoxicity [14, 15]. The research reported that leonurine could alleviate the inflammatory response, mitochondrial dysfunction, and ERS in cisplatin-induced AKI [16]. TRPA1 promotes mitochondrial Ca^2+^ dysfunction and apoptosis for colorectal cancer [17]. However, it is unclear whether TRPA1 activation is associated with cisplatin-induced ERS and mitochondrial function impairment in renal tubular epithelial cells. The lipid and metabolite exchange, as well as the maintenance of mitochondrial calcium homeostasis, depend on the mitochondria-associated endoplasmic reticulum membrane (MAM), which connects the endoplasmic reticulum and mitochondria physically [18–20]. Further investigation is needed to determine whether TRPA1, which is a cation channel dependent on the presence of Ca^2+^, is linked to endoplasmic reticulum stress and dysfunction of the mitochondria.

Therefore, this study hypothesized that TRPA1 promotes cisplatin-induced renal tubular epithelial cell injury by inducing Ca^2+^ overload-endoplasmic reticulum stress-mitochondrial damage. To verify this hypothesis, this study investigated the role of TRPA1 in regulating calcium homeostasis in the MAM between endoplasmic reticulum (ER) and mitochondria in renal tubular epithelial cells to improve cisplatin-induced AKI using human renal tubular epithelial (HK-2) cells and animal model of cisplatin-induced AKI. This study will contribute to the development of new TRPA1 inhibitors for alleviating cisplatin nephrotoxicity, and provide a theoretical basis for the treatment of cisplatin-induced AKI.

## Materials and methods

### Animals and experiments design

Male C57BL/6 mice (8–10 weeks, 20–25 g) were obtained from Dashuo Animal Center (Chengdu, Sichuan, China). The mice were subjected to a 12 h/12 h day/night cycle, and provided ad libitum access to food and water. They were allowed to acclimate to their new environment for a week before starting the study. The Animal Ethics Committee of West China Hospital (No. 20230328001) approved this study. Randomization and blinding for animal studies followed the ARRIVE guidelines for animal studies [21]. Upon completion of the acclimation period, the mice were randomly divided into 3 groups (*n* = 6): control group, model group (cisplatin), and model+TRPA1 inhibitor group (cisplatin+HC-030031). Cisplatin and HC-030031 were purchased from Sigma-Aldrich (Merck, Germany). The model group received a single intraperitoneal injection of cisplatin (20 mg/kg) [22]; the model+TRPA1 inhibitor group received a single intraperitoneal injection of cisplatin (20 mg/kg) followed by gavage administration of the TRPA1 inhibitor HC-030031 (10 mg/kg) [23]; the control group was injected with equal amount of saline containing 5% DMSO intraperitoneally. 72 h after cisplatin injection, blood was collected intraperitoneally to isolate serum [24]. All mice were euthanized with chloral hydrate (3 mL/kg, i.p.). Once euthanized, kidney tissue samples were immediately collected and stored at − 80 °C for further analysis. Serum was taken to determine blood urea nitrogen (BUN) and serum creatinine (Scr).

### Cell culture and treatment

Human renal tubular epithelial (HK-2) cells (CL-0109, Procell, Wuhan, China) were cultured in MEM medium (PM150410, Procell) supplemented with 10% fetal bovine serum (FBS) (P20522, TRAN), 100 U/mL penicillin (Sigma, Germany) and 100 g/mL streptomycin (Sigma, Germany). The medium was incubated at 37 °C and 5% to provide an optimal growth environment for the cells. Additionally, the passage number of the cells was monitored, and cells were used up to passage number 3. HK-2 cells were plated in 6-well culture plates at 3 × 10^5^ cells per well.

To study the inhibitory effect of TRPA1 inhibitor on cisplatin-induced endoplasmic reticulum stress in renal tubular epithelial cells, the experiment was conducted using HK-2 cells. HK-2 cells were randomly divided into three groups (*n* = 6): Control, cisplatin, and cisplatin+HC-030031 groups. The control group was treated with same volume of phosphate-buffered saline (PBS) for 48 h; the cisplatin group was induced with 29.16 µM cisplatin for 48 h; the cisplatin+HC-030031 group was induced with both 29.16 µM cisplatin and 30 µM HC-030031 for 48 h; dose reference to previous studies [13, 25].

To study the effects of TRPA1 activation on renal tubular epithelial cell injury and endoplasmic reticulum stress, HK-2 cells were randomly divided into four groups (*n* = 6): control, TRPA1 agonists, TRPA1 agonists+ERS inhibitor, and TRPA1 agonists+GRP75 inhibitor groups. The control group was treated with same volume of PBS for 48 h; the TRPA1 agonists group was induced with 100 µM AITC (Sigma-Aldrich, Germany) for 24 h; the TRPA1 agonists+ERS inhibitor group was treated with 1 mM 4-PBA (Sigma-Aldrich, Germany); the TRPA1 agonists+GRP75 inhibitor group was induced with both 100 µM AITC and 1 µM MKT077 (MedChemExpress, USA) for 24 h.

### Hematoxylin–eosin (H&E) staining

Kidney tissue sections were dewaxed to water and stained with hematoxylin for a duration of 20 min. Subsequently, the sections were placed in warm water at 50 °C until a blue color appeared. Afterward, the sections were stained with eosin for 5 min, and sealed with neutral gum. Finally, the sections were observed using an electron microscope (Motic China Group Co., Ltd, China) at magnifications of 100× and 400×.

### TUNEL staining

The (TdT-mediated dUTP Nick-End Labeling (TUNEL) Apoptosis Detection Kit (No. 40306ES20, Beyotime, China) was utilized to identify and quantify apoptosis in renal tissue. Kidney tissue sections were dehydrated, embedded, and dewaxed to water. Subsequently, the sections were subjected to microwave repair for 8 min using citric acid, and washed 3 times with PBS. The sections were then incubated in the dark with fluorescent TUNEL incubation solution at 37 °C for 1 h. After PBS washing, the nuclei were stained with DAPI for 15 min. Finally, the sections were scanned using a digital section scanner (3DHISTECH, Hungary) and analyzed. Representative TUNEL staining images showed DAPI in blue and TUNEL staining in green.

### Transmission electron microscope (TEM)

To observe the mitochondrial damage and morphological changes of kidney tissue and HK-2 cells, a series of steps were taken using transmission electron microscopy. Initially, the samples were prefixed with 3% glutaraldehyde and then refixed with 1% osmium tetroxide; dehydrated with acetone step by step, and embedded with Epon 812 (No. SPI-02660, ShangHai Rebiosci Biotech Co., Ltd, China). Prepared 60 nm sections by an ultramicrotome; stained with uranyl acetate for 10 min, followed by lead citrate for 2 min at room temperature. Finally, JEM-1400FLASH transmission electron microscope (JEOL, Japan) was employed for the acquisition of images. The mitochondria, autophagy, lipid droplets, the rough endoplasmic reticulum, and MAM structure were observed in detail.

### Immunofluorescence

The expressions of ERS marker proteins glucose-regulated protein 78 (GRP78), glucose-regulated protein 75 (GRP75), and C/EBP homologous protein (CHOP) in HK-2 cells were detected by immunofluorescence. Tissue slides were dewaxing, rehydrated, antigen retrieval, and blocking. The rehydrated paraffin sections (4 μm) were washed with PBS, anti-CHOP (1:100, PA5-104528, Thermo Fisher Scientific, USA), GRP75 (1:100, ab227215, abcam, UK), and GRP78 (1:200, GB11098, Servicebio, China) were incubated overnight at 4 °C, and then stained with FITC-labeled goat anti-rabbit IgG (1:100, GB22303, GB21301, GB22303, Servicebio, China) for 30 min. Nuclei were incubated with DAPI for 10 min at room temperature. Finally, the sections were analyzed by a confocal laser microscope (Olympus Corporation, Japan). The antibodies against GRP78, GRP75, and CHOP exhibited a strong green fluorescence indicating positive expression, while DAPI staining appeared as blue fluorescence representing the cell nuclei.

### Cell viability assay

The CCK-8 assay was used to detect cell viability. HK-2 cells at logarithmic growth stage were taken and inoculated in 96-well plates at a cell density of 4 × 10^4^/mL. Then, the plates were placed in a constant temperature incubator set at 37 °C and 5% CO_2_. The supernatant was discarded, and new medium (100 µL) containing CCK8 reagent (10 µL) (Biosharp, China) was added; the medium was gently shaken several times and incubated at 37  °C and 5% CO_2_ for 2 h. The absorbance value of each well was measured at 450 nm using an enzyme marker (molecular devices, Germany).

### Flow cytometry

HK-2 cell apoptosis was evaluated by flow cytometry. HK-2 cells at logarithmic growth stage were adjusted to 3 × 10^5^/well, then inoculated in 6-well plates, and incubated at 37 °C and 5% CO_2_ at constant temperature. The supernatant was aspirated and washed with PBS. Digest with trypsin and centrifuge at 250×*g* for 5 min to obtain cell precipitate. Add PBS wash again and then centrifuge. The cell suspension was then incubated in the dark for 15 min at room temperature, allowing for the Annexin V-APC (5 µL) and PI (5 µL) to bind to the appropriate cellular components Cell apoptosis was detected by flow cytometry (Beckman, Germany).

### Western blotting

The expression levels of GRP78, GRP75, CHOP, optic atrophy 1 (OPA1), mito-fusion 1 (MFN1), mito-fusion 2 (MFN2), dynamin-related protein 1 (DRP1), p-DRP1, and mitochondrial fission factor (MFF) proteins were detected using Western blotting. The total proteins in kidney tissue and HK-2 cells were extracted by RIPA lysate (Servicebio, China), and the supernatant was centrifuged to determine the protein concentration using the BCA protein quantification kit (No. P0009, Beyotime, China). The proteins were denatured by incubation at 95 °C for 15 min, separated by 10% SDS-PAGE gel, and then transferred to polyvinylidene difluoride (PVDF) membranes. PVDF membranes were placed in 5% skim milk diluted with TBST Buffer and incubated for 2 h. Antigen retrieval was performed using antigen retrieval reagent (DAKO, S2369) before blocking. Subsequently, PVDF membranes were placed in primary antibody and incubated overnight at 4 °C, and then placed in secondary antibody and incubated at room temperature for 2 h. These antibodies were used for Western blotting according to previous methods [26, 27]. The details of the antibodies used can be found in Table 1. After washing with TBST, ECL luminescent solution was added and the bands were scanned using a chemiluminescent gel imager (Shanghai Tanon Technology Co., Ltd, China).

**Table 1:** Information about antibodies for Western blotting

Antibody	Catalog numbers	Lot numbers	Host species	Source	Dilution concentration	Category
Anti-C/EBP-homologous protein (CHOP)	A0221	No. 5500017089	Rabbit	Abclonal	1:2000	Primary antibody
Anti-glucose regulated protein 75 (GRP75)	A11256	No. 4000000566	Rabbit	Abclonal	1:2000	Primary antibody
Anti-glucose regulated protein 78 (GRP78)	A0241	No. 1152920101	Rabbit	Abclonal	1:2000	Primary antibody
Anti-optic Atrophy 1 (OPA1)	A9833	No. 5500022367	Rabbit	Abclonal	1:2000	Primary antibody
Anti-mitofusin gene 1 (MFN1)	A9880	No. 5500018775	Rabbit	Abclonal	1:2000	Primary antibody
Anti-mitofusin gene 2 (MFN2)	A19678	No. 4000000157	Rabbit	Abclonal	1:2000	Primary antibody
Anti-dynamin-related protein 1 (DRP1)	A2586	No. 3560323010	Rabbit	Abclonal	1:2000	Primary antibody
Anti-phospho-dynamin-related protein 1 (p-DRP1)	ab193216	No. GR3224482-35	Rabbit	Abcam	1:1000	Primary antibody
Anti-mitochondrial fission factor (MFF)	A8700	No. 4000001243	Rabbit	Abclonal	1:2000	Primary antibody
Anti-β-actin	AC026	No. 3522101701	Rabbit	Abclonal	1:50,000	Primary antibody
Goat anti-rabbit IgG (H+L)	S0001	No. 56j9958	Goat	Affinity	1:5000	Secondary antibody

### Ca^2+^ concentration determination

Fluo-2/AM was used to measure the cellular Ca^2+^ concentration. HK-2 cells were cultured in 6-well plates in 3 × 10^5^/well. Prior to the experiment, the cells were gently washed with PBS and then centrifuged at 250×*g* for 5 min to obtain cell pellets. Incubate with Fluo 2-AM solution at 37 °C in the dark for 40 min. Wash twice with PBS, and then resuspend in 300 µL fluo-2/AM diluent for 10 min at 37 °C. Finally, cellular Ca^2+^ concentration was measured by flow cytometry, which detected the fluorescence emitted by the Fluo-2/AM dye. Similarly, kidney tissue was ground with a grinder to obtain a cell suspension; and added red blood cell lysate for 5 min, washed twice with PBS, and the cell pellet was collected by centrifugation. After the staining process, the Ca^2+^ concentration in the kidney tissue was measured using the same flow cytometry technique as described earlier.

### JC-1 assay

The JC-1 assay, a commonly used method to measure mitochondrial membrane potential, was performed in this study. HK-2 cells were inoculated in 6-well plates at a density of 3 × 10^5^/well, and incubated at 37 °C with 5% CO_2_. The JC-1 working solution was prepared by mixing 900 µL terilized deionized water, 2 µL JC-1 (500×), and 100 µL 10× incubation buffer. The cells were then incubated at 37 °C with 5% CO_2_ for 20 min. After centrifuging the cells at 350×*g* for 5 min, the supernatant was aspirated and washed twice with 1× incubation buffer. Finally, the cells were resuspended in 500 µL 1× incubation buffer for analysis.

### Mitochondrial ATP detection

The mitochondrial ATP content of HK-2 cells was determined using the ATP content assay kit (A095-1-1, Nanjing Jiancheng Bioengineering Institute, China). HK-2 cell culture supernatant was separated by centrifugation, and the cell were pelleted and broken to determine the protein concentration. In order to extract ATP from the mitochondria, the cells were heated in a boiling water bath for 10 min, and vortexed for 1 min. After standing for 5 min at room temperature, the absorbance values were measured at 636 nm.

### Intracellular ROS levels determination

The level of ROS in HK-2 cells was detected according to the instructions of the Human ROS ELISA KIT kit (ZCIBIO Technology Co., Ltd, China), and the OD value of each well was measured at 450 nm.

### Statistical analysis

SPSS 17.0 statistical software (SPSS Inc., USA) was utilized to conduct statistical analysis in this study. The Shapiro–Wilk method was used to test for normal distribution, and Levene’s test was used for homogeneity of variance analysis. One-Way ANOVA test was used for comparison between multiple sample means, and the LSD test (homogeneity of variance) or Tamhane’s T2 test (heterogeneity of variance) was performed. All data are represented as box plots with minimum to maximum, showing all points. The results were represented as mean ± standard deviation (SD), and statistical significance was determined when the *p*-value was less than 0.05, indicating significant differences between the groups being compared.

## Results

### TRPA1 inhibitor HC-030031 protects cisplatin-induced AKI in mice

**Fig. 2:**
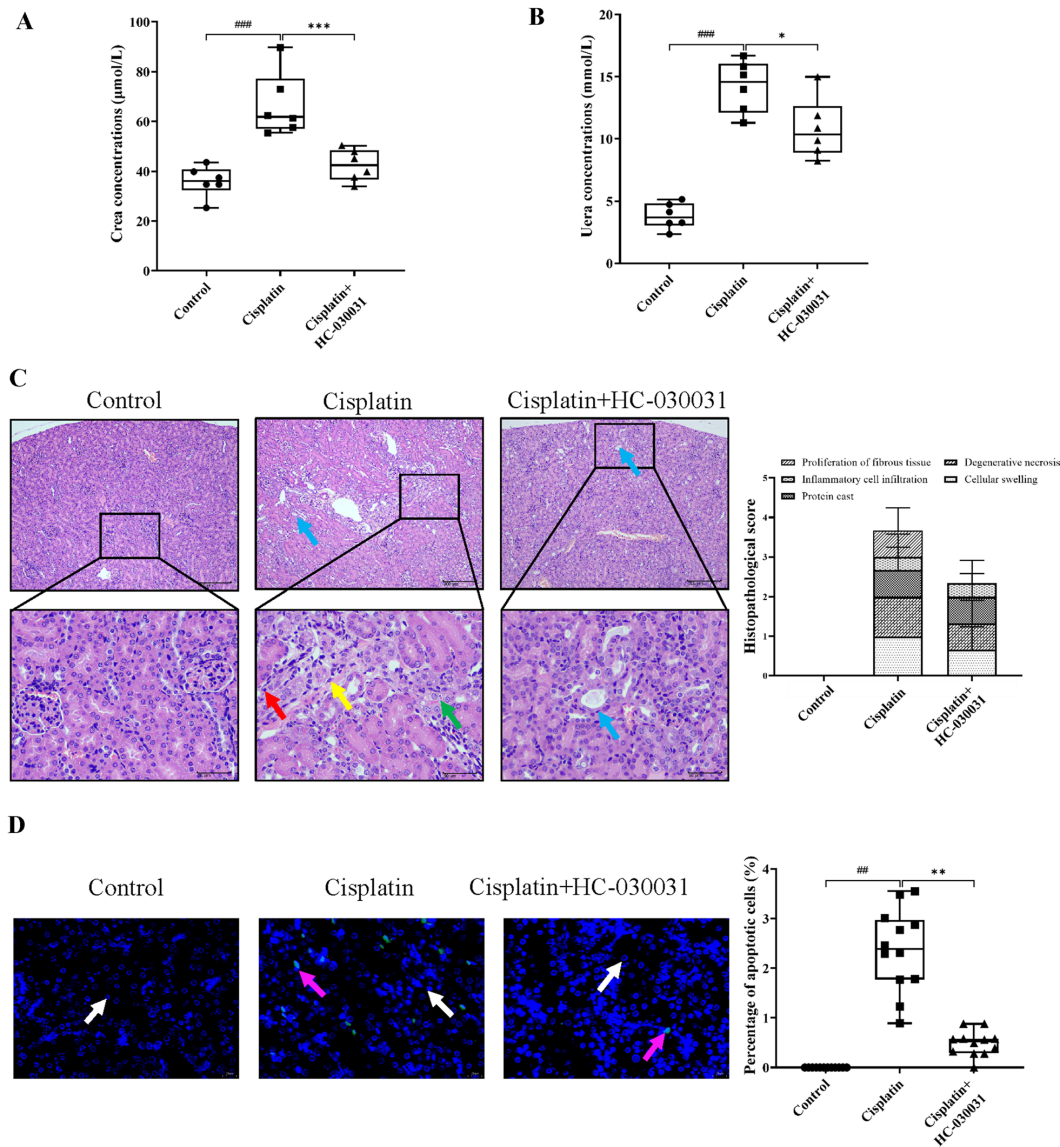
HC-030031 ameliorates cisplatin-induced kidney function, histopathological damage, and apoptosis in AKI mice. HC-030031 reduced Scr (**A**) and BUN (**B**) concentrations in serum. **C** H&E staining was used to observed pathological changes of the kidney (*n* = 3 mice per experimental group). A 4-point scale was used for scoring, with no lesions recorded as 0, slight as 1, mild as 2, moderate as 3, and severe as 4. Renal tubular epithelial degeneration necrosis (green arrows) lymphocytes (red arrows), fibroblasts (yellow arrows), and protein tubular pattern (blue arrows) are shown in the figure. Scale bar = 50 and 200 μm. **D** Renal cell apoptosis was observed by TUNEL fluorescence staining. Blue light represents DAPI, green light represents Tunel, white arrows represent normal cells, and purple arrows represent apoptotic cells. Scale bar = 20 μm. The results were shown as means ± SD. ^##^*P* < 0.01, ^###^*P* < 0.001, compared with the control group; *P < 0.05, **P < 0.01, ***P < 0.001, compared with the cisplatin group

To investigate whether TRPA1 inhibitor HC-030031 has a protective effect in cisplatin-induced AKI, we evaluated renal function indicators (Scr and BUN), histopathological changes and apoptosis in the kidney. As shown in Fig. 2A, B, compared with the control group, Scr and BUN levels in the cisplatin group were considerably elevated (both *P* < 0.001), whereas the cisplatin+HC-030031 group significantly reduced the cisplatin-induced Scr and BUN levels (*P* < 0.001, *P* < 0.05). H&E staining revealed some pathological changes in the cisplatin group, which were characterized by localized tubular epithelial cell swelling, degenerative necrosis, protein tubular pattern seen in some tubular lumens, a small amount of inflammatory cell infiltration, and a small amount of fibrous tissue hyperplasia. HC-030031 treatment greatly alleviated the pathological damage of the kidney (Fig. 1C). Subsequently, we evaluated the effect of HC-030031 on cell apoptosis in the kidney tissue of cisplatin injected mice by TUNEL staining. The findings indicated that TUNEL-positive cells were not found in normal cells, but were significantly found in the kidney tissues of the cisplatin group (*P* < 0.01). HC-030031 treatment significantly reduced the percentage of positive expression of apoptotic cells (*P* < 0.01) (Fig. 2D). Taken together, our results suggest that TRPA1 inhibitor HC-030031 ameliorates cisplatin-induced kidney function, histopathological damage, and apoptosis in AKI mice.

### TRPA1 inhibitor HC-030031 protects endoplasmic reticulum stress-mitochondrial damage in cisplatin-induced AKI in mice

**Fig. 3:**
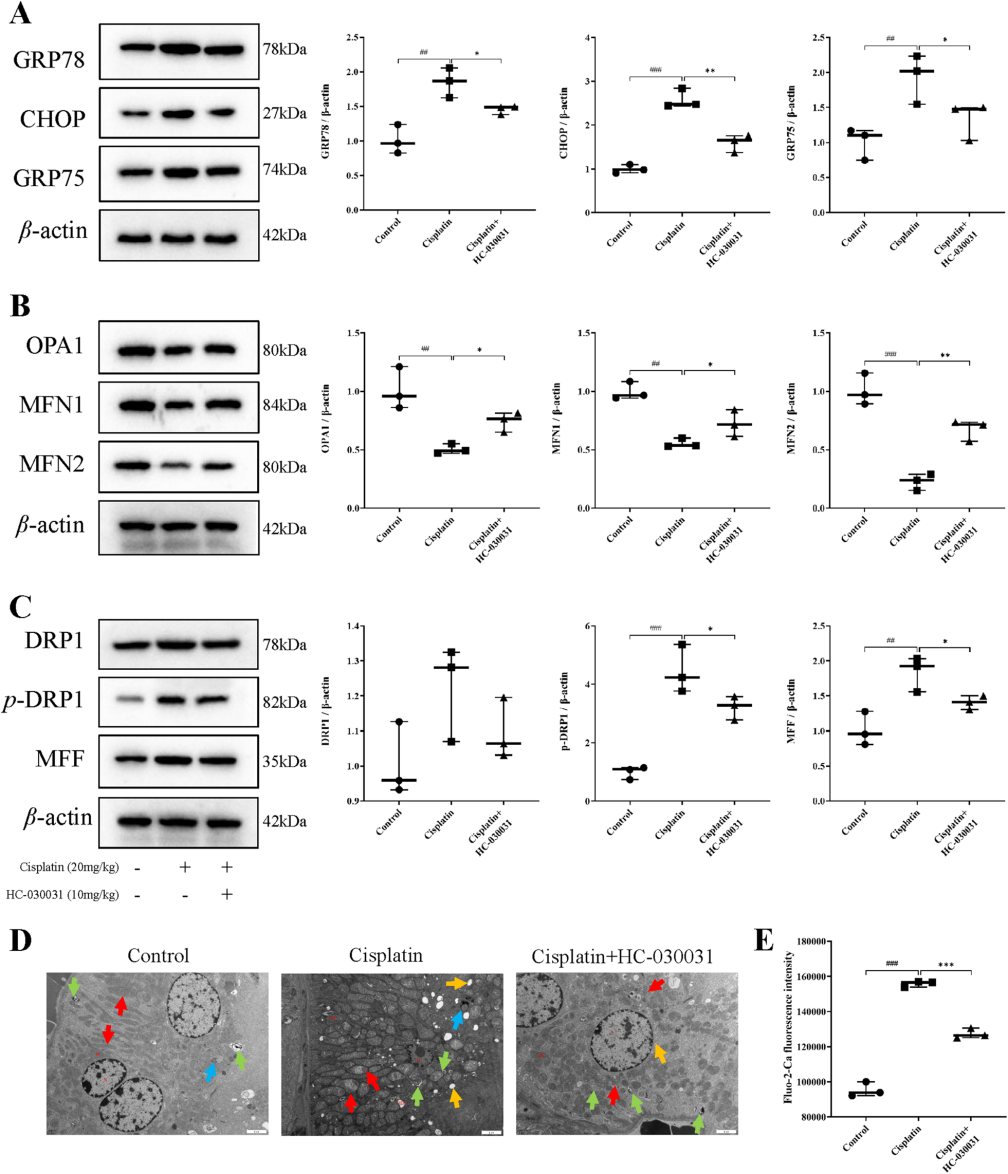
HC-030031 protects endoplasmic reticulum stress-mitochondrial damage in DP-induced AKI in mice. **A** The ERS-related proteins GRP78, CHOP, and GRP75 levels were measured using western blot. **B** The levels of mitochondrial fusion proteins OPA1, MFN1, and MFN2 were analyzed using western blot. **C** The expression levels of mitochondrial division proteins DRP1, p-DRP1 and MFF were analyzed using western blot. Representative images of Western blotting are shown on the left, quantitative results of Western blotting are shown on the right. **D** HC-030031 improved mitochondrial damage in the kidney. Mitochondria are represented by red arrows, autophagy is represented by green arrows, lipid droplets are represented by blue arrows, and the rough endoplasmic reticulum is represented by yellow arrows. Scale bar = 2 μm. **E** HC-030031 decreased intracellular calcium ion concentration. The western blot results were exhibited after being normalized to β-actin. The results were shown as means ± SD, *n* = 3. ^##^*P* < 0.01, ^###^*P* < 0.001, compared with the control group; *P < 0.05, **P < 0.01, compared with the cisplatin group

To investigate whether TRPA1 expression is associated with ERS and mitochondrial morphological dysfunction during cisplatin-induced AKI in mice, we examined the expression levels of ERS, mitochondrial morphology (division/fusion) related proteins using western blotting, observed mitochondrial damage and morphological changes by TEM, and measured the intra-mitochondrial calcium ion concentration using the Fluo-2 AM calcium ion probe. As shown in Fig. 3A, the expression levels of ERS-related proteins GRP78, CHOP, and GRP75 were significantly elevated in the cisplatin group (*P* < 0.01, *P* < 0.001, *P* < 0.01), while they were significantly reduced in the cisplatin+HC-030031 group (*P* < 0.05, *P* < 0.01, *P* < 0.05). As shown in Fig. 3B, the expression levels of mitochondrial fusion-related proteins OPA1, MFN1, and MFN2 were reduced in the kidneys of mice with cisplatin-induced AKI (*P* < 0.01, *P* < 0.01, *P* < 0.001), but HC-030031 reversed these levels (*P* < 0.05, *P* < 0.05, *P* < 0.01). As shown in Fig. 3C, the expression levels of mitochondrial division-related proteins p-DRP1 and MFF were increased in the cisplatin group (*P* < 0.001, *P* < 0.01), while the expression of the proteins was decreased after HC-030031 treatment (both *P* < 0.05). TEM results showed that the mitochondrial morphological structure of epithelial cells in kidney tissue was obviously abnormal in the cisplatin group; the mitochondria were swollen, cristae were dissolved and broken, matrix particles were reduced; the rough endoplasmic reticulum was expanded in a vesicle-like structure; lipid droplets and autophagy were seen in the cytoplasm. However, HC-030031 improved mitochondrial damage in the kidney (Fig. 3D). In addition, it was confirmed that cisplatin treatment enhanced intracellular calcium ion concentration, while HC-030031 treatment decreased intracellular calcium ion concentration (both *P* < 0.05) (Fig. 3E). The above results suggested that TRPA1 may have the effect of mediating cisplatin-induced AKI in mice, while inhibition of TRPA1 may protect kidneys from ERS-mitochondrial injury.

### TRPA1 inhibitor ameliorates cisplatin-induced ERS and mitochondrial damage in renal tubular epithelial cells

#### TRPA1 inhibitor HC-030031 inhibits cisplatin-induced ERS in renal tubular epithelial cells

**Fig. 4:**
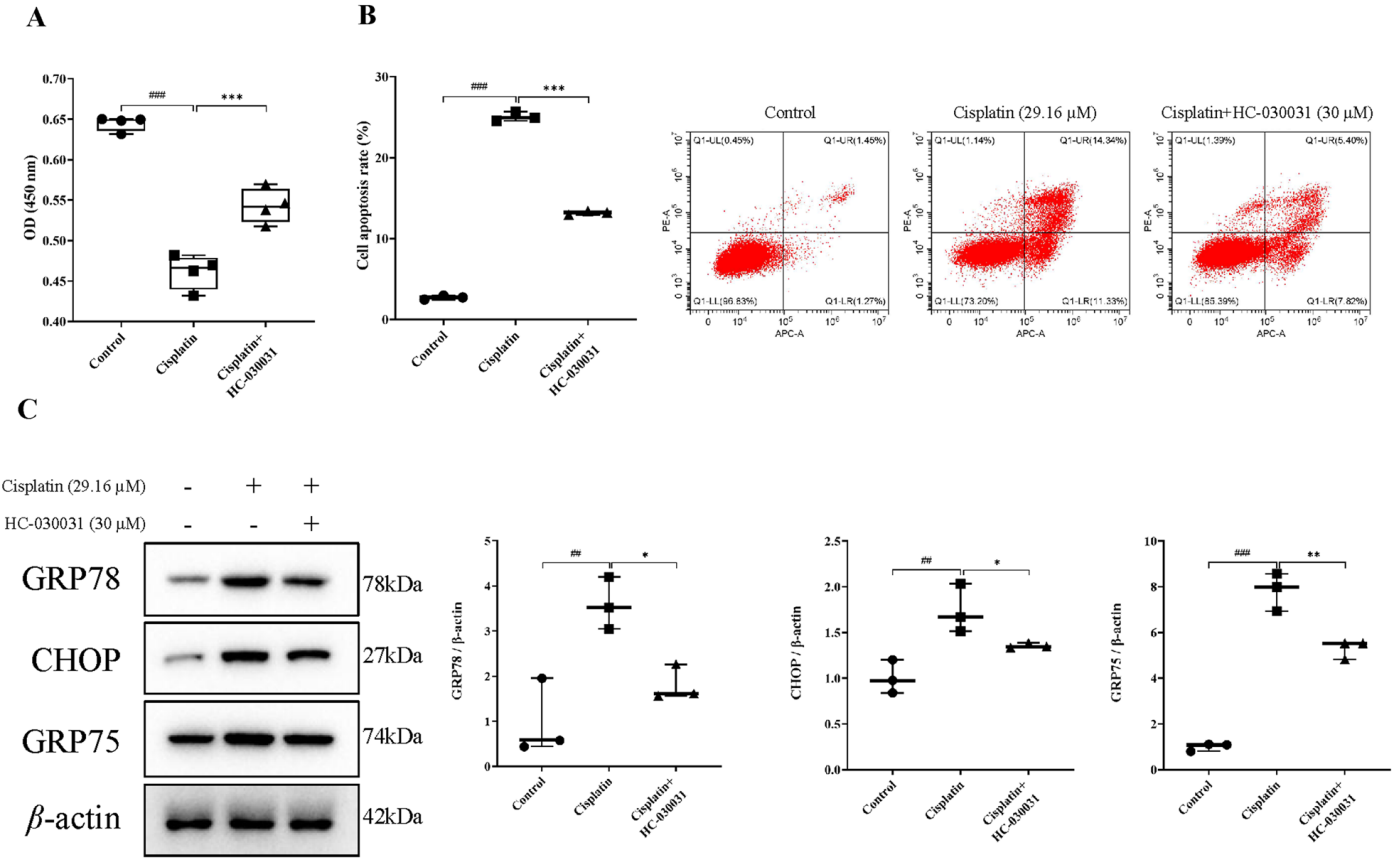
HC-030031 inhibits cisplatin-induced ERS in renal tubular epithelial cells. **A** Cell proliferation activity was detected by CCK8 assay (*n* = 4). **B** The apoptotic of the HK-2 cells was determined by flow cytometry. **C** The ERS-related proteins GRP78, CHOP, and GRP75 levels in HK-2 cells were measured using western blot. The results were shown as means ± SD (*n* = 3). ^##^*P* < 0.01, ^###^*P* < 0.001, compared with the control group; *P < 0.05, **P < 0.01, compared with the cisplatin group. The control group was left untreated; the cisplatin group was induced with 29.16 µM cisplatin for 48 h; the cisplatin+HC-030031 group was induced with both cisplatin and 30 µM HC-030031 for 48 h

We have previously demonstrated the protective effect of TRPA1 inhibitor HC-030031 on AKI in mice. Then we established a cisplatin-induced renal tubular epithelial cell injury model at the cellular level to further analyze the mechanism of TRPA1-triggered ERS during cisplatin-induced renal tubular epithelial cell injury. CCK-8 results showed that HC-030031 treatment increased the cisplatin-induced decrease in proliferative activity of HK-2 cells (*P* < 0.05) (Fig. 4A). Additionally, HC-030031 treatment significantly attenuated cisplatin-induced apoptosis in HK-2 cells (*P* < 0.05) (Fig. 4B). Furthermore, we used western blotting to detect the expression levels of ERS marker proteins GRP78, GRP75, and CHOP. The results showed that cisplatin induced an increase in the expression of GRP78, GRP75, and CHOP (*P* < 0.01, *P* < 0.01, *P* < 0.001), while HC-030031 decreased their expression (*P* < 0.05, *P* < 0.05, *P* < 0.01) (Fig. 4C). In short, HC-030031 had an inhibitory effect on cisplatin-induced ERS in renal tubular epithelial cells.

#### TRPA1 inhibitor HC-030031 ameliorates cisplatin-induced mitochondrial damage and calcium overload in renal tubular epithelial cells

**Fig. 5:**
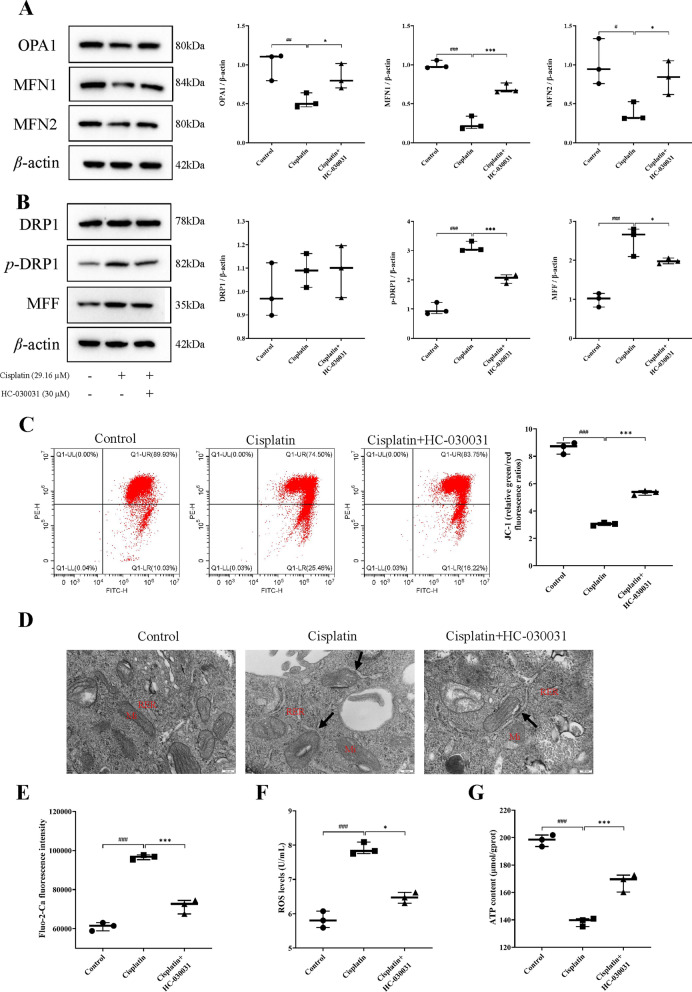
HC-030031 ameliorates cisplatin-induced mitochondrial damage and calcium overload in renal tubular epithelial cells. **A** The levels of mitochondrial fusion proteins OPA1, MFN1, and MFN2 were analyzed using western blot. **B** The expression levels of mitochondrial division proteins DRP1, p-DRP1 and MFF were analyzed using western blot. **C** Mitochondrial membrane potential of cell was detected by JC-1 assay. **D** The morphological changes of mitochondria were observed by TEM. *Mi* mitochondria, *RER* rough endoplasmic reticulum; the black arrows represent MAM. Scale bar = 200 nm. **E** Fluo-2/AM was used to measure Ca^2+^ concentration in HK-2 cells using flow cytometry. **F** Intracellular ROS levels were detected using an ELISA kit at 450 nm. **G** The mitochondrial ATP content was determined using the ATP content assay kit at 636 nm. The results were shown as means ± SD (*n* = 3). ^#^*P* < 0.05, ^##^*P* < 0.01, ^###^*P* < 0.001, compared with the control group; *P < 0.05, ***P < 0.001, compared with the cisplatin group

Then we investigated the relationship between TRPA1 expression and mitochondrial morphological dysfunction and calcium overload during cisplatin-induced renal tubular epithelial cell injury. Western blotting was used to detect the expression levels of mitochondrial fusion (OPA1, MFN1, and MFN2) and division (DRP1, p-DRP1, and MFF) proteins. The results indicated that HC-030031 increased the cisplatin-induced decrease in the expression of mitochondrial fusion proteins (OPA1, MFN1, and MFN2) (*P* < 0.05, *P* < 0.001, *P* < 0.05) (Fig. 5A), and decreased the cisplatin-induced increase in the expression of mitochondrial division proteins (p-DRP1 and MFF) (*P* < 0.001, *P* < 0.05) (Fig. 5B). This result was consistent with the results of the AKI mouse model. The mitochondrial membrane potential of HK-2 cells was detected using the JC-1 assay. As shown in Fig. 5C, HC-030031 treatment significantly elevated the mitochondrial membrane potential of HK-2 cells compared with the cisplatin-treated group (*P* < 0.001). In addition, TEM results showed that HC-030031 alleviated cisplatin-induced mitochondrial morphological damage. There were MAM structures between mitochondria and rough endoplasmic reticulum (RES) in the cisplatin group, while HC-030031 may reduce the MAM area. (Fig. 5D). Fluo-2 AM calcium probe results showed that calcium ion concentration was increased in the cisplatin group (*P* < 0.001), while inhibition of TRPA1 decreased the calcium ion concentration (*P* < 0.001) (Fig. 5E). Moreover, the TRPA1 inhibitor HC-030031 also reduced the cisplatin-induced up-regulation of intracellular ROS levels (*P* < 0.05), and increased cisplatin-induced down-regulation of mitochondrial ATP (*P* < 0.001) (Fig. 5F, G). In summary, TRPA1 inhibitors had an ameliorative effect on cisplatin-induced mitochondrial morphological and functional impairment and Ca^2+^ overload in renal tubular epithelial cells.

### TRPA1 promotes mitochondrial dysfunction in renal tubular epithelial cells through MAM

#### TRPA1 agonists promotes renal tubular epithelial cell injury and ERS, while ERS inhibitors and GRP75 inhibitors ameliorate cell injury

**Fig. 6:**
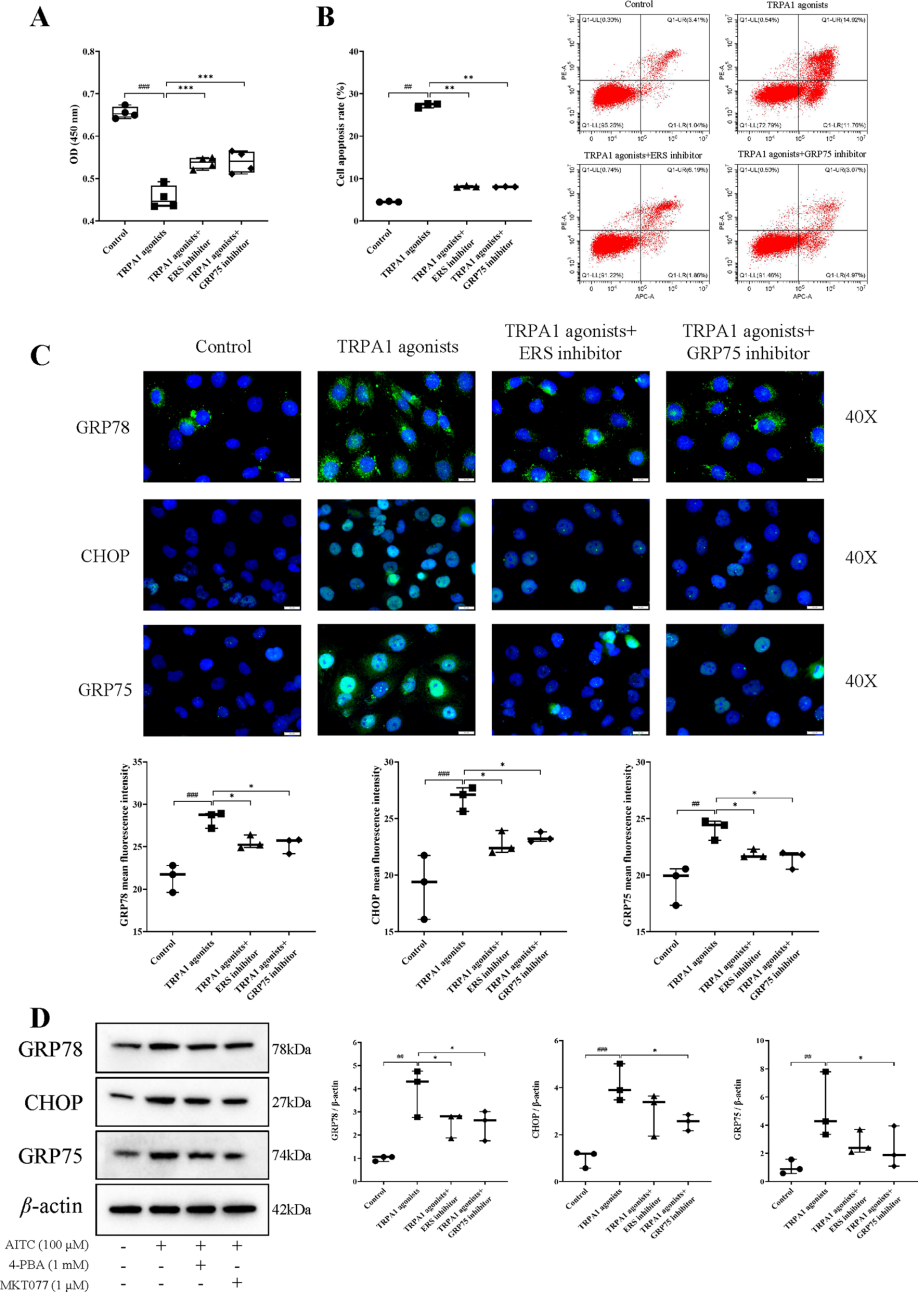
TRPA1 agonists promote renal tubular epithelial cell injury and ERS, while ERS inhibitors and GRP75 inhibitors ameliorate cell injury. **A** Cell proliferation activity of HK-2 cell was detected by CCK8 assay. **B** The apoptotic of the HK-2 cells was determined by flow cytometry. **C** ERS-related proteins GRP78, GRP75 and CHOP in HK-2 cells were detected by immunofluorescence staining. Scale bar = 20 μm. **D** The ERS-related proteins GRP78, CHOP, and GRP75 levels in HK-2 cells were measured using western blot. The results were shown as means ± SD (*n* = 3). ^##^*P* < 0.01, ^###^*P* < 0.001, compared with the control group; *P < 0.05, **P < 0.01, ***P < 0.001, compared with the cisplatin group. Control: left untreated; TRPA1 agonists: 100 µM AITC for 24 h; TRPA1 agonists+ERS inhibitor: 100 µM AITC and 1 mM 4-PBA for 24 h; TRPA1 agonists+GRP75 inhibitor: 100 µM AITC and 1 µM MKT077 for 24 h

The effects of HC-030031 on ER and mitochondrial damage have been verified previously, respectively. However, whether TRPA1-promoted ERS mediates mitochondrial morphology and dysfunction via endoplasmic reticulum-mitochondrial coupling (MAM) deserves further investigation. The CCK-8 results showed that TRPA1 agonists (AITC) decreased the proliferative activity of HK-2 cells, whereas ERS inhibitor (4-PBA) and GRP75 inhibitor (MKT077) treatment significantly increased the proliferative activity of the cells (both *P* < 0.001) (Fig. 6A). In addition, we evaluated the role of TRPA1 agonists in apoptosis. In Fig. 6B, TRPA1 agonists induced apoptosis in HK-2 cells, which was ameliorated by ERS inhibitor and GRP75 inhibitor (both *P* < 0.01). Furthermore, we detected ERS-tagged proteins GRP78, GRP75, and CHOP expression by western blotting as well as immunofluorescence staining. As shown in Fig. 6C, D, GRP78, GRP75 and CHOP expression were elevated in the TRPA1 agonists group compared with the control group (*P* < 0.001, *P* < 0.001, *P* < 0.01); while compared with the TRPA1 agonists group, the expression of GRP78, GRP75 and CHOP was decreased in the TRPA1 agonists and TRPA1 agonists+ERS inhibitor groups (both *P* < 0.05). The results further suggest that TRPA1 damage to renal tubular epithelial cells is associated with triggering ERS.

#### TRPA1 agonists mediated mitochondrial calcium overload promotes mitochondrial morphological damage and dysfunction in renal tubular epithelial cells, while ERS inhibitors and GRP75 inhibitors ameliorate cell injury

**Fig. 7:**
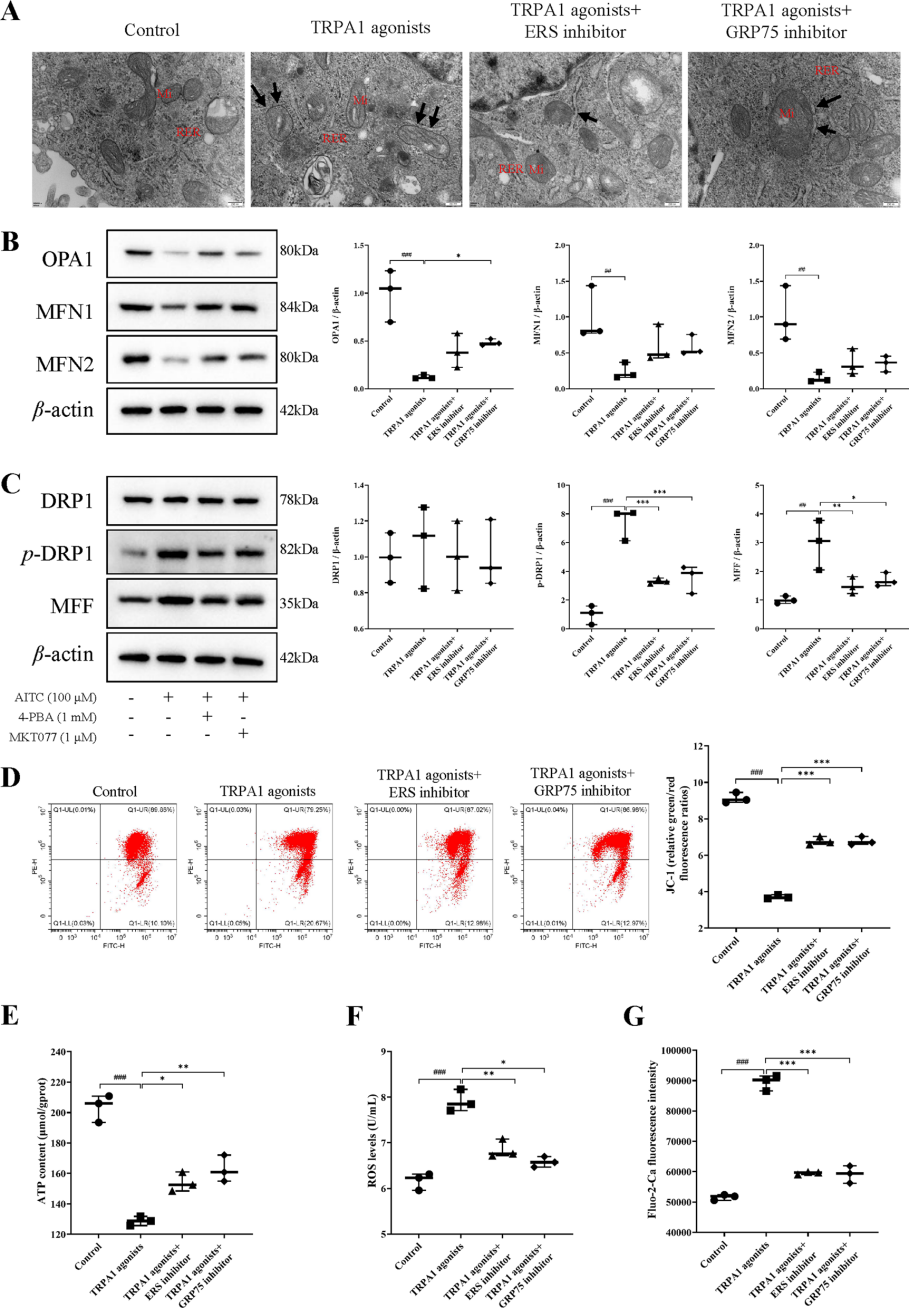
TRPA1 agonists promote mitochondrial morphological damage and dysfunction in renal tubular epithelial cells, while ERS inhibitors and GRP75 inhibitors ameliorate cell injury. **A** The morphological changes of mitochondria were observed by TEM. *Mi* mitochondria, *RER* rough endoplasmic reticulum; the black arrows represent MAM. Scale bar = 200 nm. **B** The levels of mitochondrial fusion proteins OPA1, MFN1, and MFN2 were analyzed using western blot. **C** The expression levels of mitochondrial division proteins DRP1, p-DRP1 and MFF were analyzed using western blot. **D** Mitochondrial membrane potential of HK-2 cell was detected by JC-1 assay. **E** ERS inhibitors and GRP75 inhibitors increased cisplatin-induced down-regulation of mitochondrial ATP. **F** ERS inhibitors and GRP75 inhibitors reduced the cisplatin-induced up-regulation of intracellular ROS levels. **G** ERS inhibitors and GRP75 inhibitors reduced the cisplatin-induced up-regulation of Ca^2+^ concentration in HK-2 cells. The results were shown as means ± SD (*n* = 3). ^##^*P* < 0.01, ^###^*P* < 0.001, compared with the control group; *P < 0.05, **P < 0.01, ***P < 0.001, compared with the cisplatin group. Control: left untreated; TRPA1 agonists: 100 µM AITC for 24 h; TRPA1 agonists+ERS inhibitor: 100 µM AITC and 1 mM 4-PBA for 24 h; TRPA1 agonists+GRP75 inhibitor: 100 µM AITC and 1 µM MKT077 for 24 h

MAM was used to indicate the interaction between the endoplasmic reticulum and the mitochondria. The number and morphological changes of cellular mitochondria and MAM were observed by TEM. The results showed that TRPA1 agonists promoted mitochondrial morphological damage and increased MAM area, while ERS inhibitor and GRP75 inhibitor decreased cellular mitochondrial damage and MAM area (Fig. 7A). Western blotting was used to detect mitochondrial fusion (OPA1, MFN1, and MFN2) and division (DRP1, p-DRP1, and MFF) protein expression. As shown in Fig. 7B, ERS inhibitor and GRP75 inhibitor elevated the TRPA1 agonists-induced decrease in OPA1, MFN1, and MFN2 protein levels. Conversely, ERS inhibitor and GRP75 inhibitor reduced DRP1, p-DRP1, and MFF protein levels elevated by TRPA1 agonists (Fig. 7C). In addition, TRPA1 agonists decreased MMP and mitochondrial ATP (both *P* < 0.001), which were significantly increased by ERS inhibitor and GRP75 inhibitor (*P* < 0.001, *P* < 0.001, *P* < 0.05, *P* < 0.01) (Fig. 7D, E). ERS inhibitor and GRP75 inhibitor also decreased TRPA1 agonists-induced increase in intracellular ROS levels (*P* < 0.01, *P* < 0.05) (Fig. 7F). By observing Ca^2+^ changes, it was found that TRPA1 agonists promoted mitochondrial calcium overload, which was significantly ameliorated by ERS inhibitor and GRP75 inhibitor (both *P* < 0.001) (Fig. 7G). The above results confirmed that TRPA1 may induce mitochondrial calcium overload by triggering ERS and up-regulating GRP75 expression, thus promoting mitochondrial morphology and dysfunction.

## Discussion

Cisplatin, as the most widely used chemotherapeutic drug in clinical practice, is particularly effective in causing tumor cell death. However, cisplatin is easy to reach cells throughout the body with blood circulation, and is often accompanied by various toxic side effects, including nephrotoxicity, neurotoxicity, ototoxicity, and drug resistance. The cisplatin-induced acute kidney injury (AKI), with a high mortality rate, is a serious public health problem. Numerous studies have shown that AKI is closely associated with oxidative stress, immune inflammation, apoptosis, mitochondrial damage, ERS, and other causes [28–30].

TRPA1, a calcium-permeable nonselective cation channel, has been reported to play a key role in various types of pain, and TRPA1 antagonists can reduce or eliminate cisplatin-induced neuropathic pain [31, 32]. Our previous studies have revealed that cisplatin promotes TRPA1 expression, and TRPA1 promotes cisplatin-induced renal tubular apoptosis through activation of the calcium-dependent NFAT/p53 signaling pathway [13]. In the present study, we demonstrated that cisplatin can cause renal tissue injury and cell apoptosis, and TRPA1 inhibitor HC-030031 can significantly alleviate kidney dysfunction, histopathological injury and cell apoptosis in mice, thus playing a protective role against kidney injury, which is consistent with the previous studies.

ERS is a protective mechanism for cells, while excessive ERS also induces apoptosis [33, 34]. ERS may exacerbate acute kidney injury by up-regulating CHOP expression, activating GRP78, and promoting the Caspase 12 apoptotic pathway [35, 36]. Mitochondrial homeostasis is required for optimal renal function; however, mitochondrial function is disrupted in AKI, which further promotes renal impairment [37]. As the most prevalent signal transduction factor in the body, calcium ions play an crucial part in cell division, growth, and death [38]. Activation of TRPA1 increases intracellular calcium influx, while internalization of calcium crystals into renal tubular cells may lead to acute kidney injury [39, 40]. Endoplasmic reticulum stress and mitochondrial malfunction have both been linked to intracellular Ca^2+^ overload, according to research [41]. ERS is accompanied by disturbed Ca^2+^ homeostasis. Ca^2+^ is released from the ER into the cytoplasm when ERS is aggravated, and the increased intracellular Ca^2+^ concentration and oxidative stress increase the Ca^2+^ concentration in mitochondria, which leads to a decrease in mitochondrial membrane potential, activation of caspase proteins, and initiation of apoptosis [42]. TRPA1, being a key Ca^2+^ homeostasis regulating channel, may cause ERS and mitochondrial dysfunction. Endoplasmic reticulum-mitochondrial coupling (MAM) is an critical structure for maintaining endoplasmic reticulum and mitochondrial homeostasis, and ERS can promote mitochondrial morphological and functional damage through GRP75-mediated Ca^2+^ overload [43]. Studies have reported that MAM serve a key function in the maintenance of cellular Ca^2+^ homeostasis. Cellular Ca^2+^ overload triggers a cascade reaction of reactive oxygen species (ROS) production, leading to decreased MMP and mitochondrial damage, and MAM promotes renal tubular cell death by mediating massive Ca^2+^ transport [44, 45]. In this study, we investigated the balance of TRPA1 regulation of endoplasmic reticulum and mitochondrial homeostasis by introducing MAM to reveal that TRPA1 mediates the cytotoxic effects of cisplatin-induced ERS promoting mitochondrial dysfunction. Furthermore, we revealed the protective effects of TRPA1 inhibitors on cisplatin-induced ERS and mitochondrial morphological dysfunction in renal tubular epithelial cells in vivo and in vitro. We also explored the mechanism of TRPA1 through inducing ERS to promote renal tubular epithelial cell injury and mitochondrial morphological dysfunction by using TRPA1 agonist combined with ERS inhibitor to induce renal tubular epithelial cells. In addition, to investigate the role of MAM in TRPA1-induced ERS promoting mitochondrial injury, we used TRPA1 agonist combined with GRP75 inhibitor to intervene in renal tubular epithelial cells. The results demonstrated that TRPA1 promotes mitochondrial morphological dysfunction by inducing endoplasmic reticulum-mitochondrial Ca^2+^ overload. Briefly, TRPA1 may promote mitochondrial morphology and dysfunction by triggering ERS and up-regulating GRP75 expression to induce mitochondrial calcium overload, whereas TRPA1 inhibitor HC-030031 protects against cisplatin-induced ERS-mitochondrial damage and reduces calcium overload in AKI. Additionally, although this study showed a pro-apoptotic effect of TRPA1, the key mechanisms by which TRPA1 regulates the ERS-mediated apoptotic pathway remain to be further investigated.

Although there are several novelty and strengths in our study, there are some limitations and outlook for future improvements. Firstly, animal models and in vitro experiments do not always reflect human pathophysiology, which raises concerns about the effect of TRPA1 on AKI from animals to humans. Secondly, While the role of TRPA1 in cisplatin nephrotoxicity is undoubtedly significant, the mechanisms of downstream signaling pathway of TRPA1 are still elusive. Additionally, although this study demonstrated a pro-apoptotic effect of TRPA1, the underlying mechanism remains to be elucidated. Future investigations could focus on delineating this pathway more clearly. To facilitate the clinical application of TRPA1, it is crucial to conduct further comprehensive studies on the off-target effects and long-term effects of HC-030031 to ensure its safety. Moreover, future research should prioritize the development of novel TRPA1 inhibitors with high specificity and low toxicity. Additionally, it is essential to delve deeper into the molecular mechanism of TRPA1’s role in mitochondrial function and ER stress. Furthermore, investigating the potential of combination therapy using cisplatin and HC-030031, including determining the optimal dosage and timing for both efficacy and safety, would be valuable areas for future studies.

**Fig. 8:**
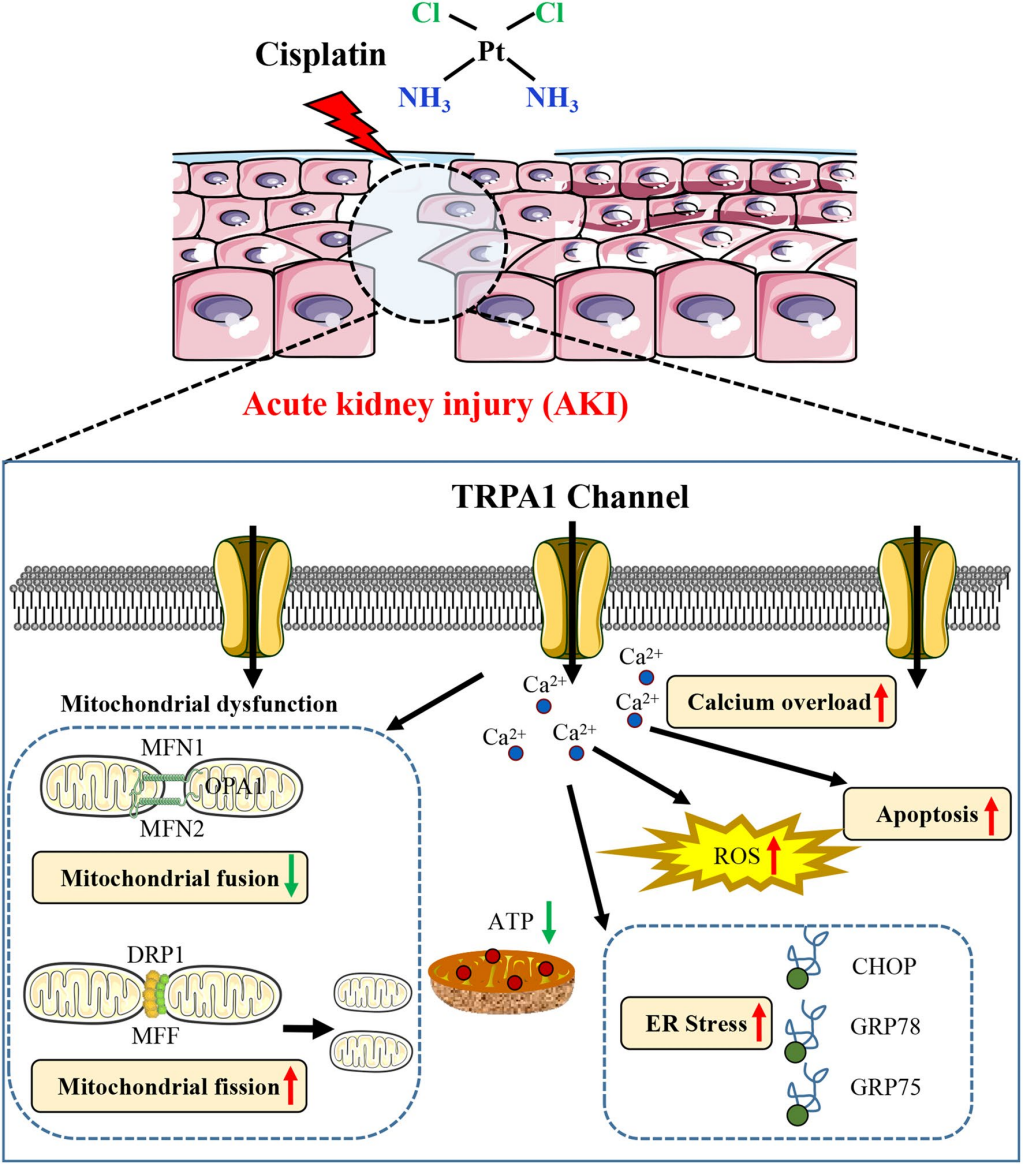
The mechanism of TRPA1 promotes acute kidney injury via regulating the endoplasmic reticulum stress and mitochondrial injury pathway. *ER* endoplasmic reticulum, *ROS* reactive oxygen species, *ATP* adenosine triphosphate

In summary, TRPA1 can promote mitochondrial morphology and dysfunction by inducing ERS to mediate cisplatin-induced renal tubular epithelial cell injury, and TRPA1 can promote mitochondrial morphology and dysfunction by triggering ERS and inducing mitochondrial calcium overload through up-regulation of GRP75 expression. This study demonstrated the mechanism of TRPA1-induced endoplasmic reticulum stress and mitochondrial damage in promoting cisplatin-induced renal tubular epithelial cell injury (Fig. 8). Furthermore, this study provides an important theoretical basis for revealing the toxic role of TRPA1 in cisplatin-induced renal tubular epithelial injury and targeting TRPA1 inhibition to improve AKI. We also linked ERS with mitochondrial injury by MAM to explore the core effect of TRPA1-induced ERS promoting mitochondrial morphology and dysfunction in mediating cisplatin nephrotoxicity, providing a new perspective for the treatment of TRPA1-mediated related cellular injury such as cisplatin-induced AKI.

## Data Availability

Not applicable.
